# Influenza surveillance capacity improvements in Africa during 2011‐2017

**DOI:** 10.1111/irv.12818

**Published:** 2020-11-04

**Authors:** Ledor S. Igboh, Meredith McMorrow, Stefano Tempia, Gideon O. Emukule, Ndahwouh Talla Nzussouo, Margaret McCarron, Thelma Williams, Vashonia Weatherspoon, Ann Moen, Derrar Fawzi, Richard Njouom, Emmanuel Nakoune, Coulibaly Dauoda, Hugo Kavunga‐Membo, Mary Okeyo, Jean‐Michel Heraud, Ivan Kiggundu Mambule, Samba Ousmane Sow, Almiro Tivane, Adamou Lagare, Adedeji Adebayo, Ndongo Dia, Vida Mmbaga, Issaka Maman, Julius Lutwama, Paul Simusika, Sibongile Walaza, Punam Mangtani, Patrick Nguipdop‐Djomo, Cheryl Cohen, Eduardo Azziz‐Baumgartner, ANISE Network Working Group

**Affiliations:** ^1^ Influenza Division National Center for Immunization and Respiratory Diseases Centers for Disease Control and Prevention Atlanta GA USA; ^2^ London School of Hygiene and Tropical Medicine London UK; ^3^ School of Public Health Faculty of Health Sciences University of Witwatersrand Johannesburg South Africa; ^4^ Influenza Program Centers for Disease Control and Prevention Pretoria South Africa; ^5^ MassGenics Atlanta GA USA; ^6^ Centers for Disease Control and Prevention Nairobi Kenya; ^7^ Centers for Disease Control and Prevention Accra Ghana; ^8^ Institut Pasteur of Algeria Algiers Algeria; ^9^ Center Pasteur du Cameroon Yaounde Cameroon; ^10^ Institut Pasteur d'Bangui Bangui Central African Republic; ^11^ National Institute Public Hygiene/Ministry of Health Abidjan Cote d'Ivoire; ^12^ Institut National de Recherche Bio‐medicale Kinshasa Democratic Republic of Congo; ^13^ National Public Health Institute Nairobi Kenya; ^14^ Virology Unit National Influenza Centre Institute Pasteur de Madagascar Antananarivo Madagascar; ^15^ Malawi‐Liverpool Wellcome Trust Clinical Research Programme Blantyre Malawi; ^16^ Central National Influenza Laboratory/Ministry of Health Bamako Mali; ^17^ Instituto Nacional de Saude Moputo Mozambique; ^18^ Center de Recherche Medicale et Sanitaire Niamey Niger; ^19^ Nigeria Centers for Disease Control Abuja Nigeria; ^20^ Institut Pasteur de Dakar Dakar Senegal; ^21^ National Reference Laboratory Dar es Salaam Tanzania; ^22^ National Influenza Reference Laboratory Lome Togo; ^23^ Uganda Virus Research Institute Entebbe Uganda; ^24^ National Influenza Center University of Zambia Teaching Hospital Lusaka Zambia; ^25^ National Influenza Center Johannesburg South Africa; ^26^ Centre for Respiratory Disease and Meningitis National Institute for Communicable Diseases Johannesburg South Africa

**Keywords:** Africa, ANISE, capacity, influenza, surveillance

## Abstract

**Background:**

Influenza surveillance helps time prevention and control interventions especially where complex seasonal patterns exist. We assessed influenza surveillance sustainability in Africa where influenza activity varies and external funds for surveillance have decreased.

**Methods:**

We surveyed African Network for Influenza Surveillance and Epidemiology **(**ANISE) countries about 2011‐2017 surveillance system characteristics. Data were summarized with descriptive statistics and analyzed with univariate and multivariable analyses to quantify sustained or expanded influenza surveillance capacity in Africa.

**Results:**

Eighteen (75%) of 24 ANISE members participated in the survey; their cumulative population of 710 751 471 represent 56% of Africa's total population. All 18 countries scored a mean 95% on WHO laboratory quality assurance panels. The number of samples collected from severe acute respiratory infection case‐patients remained consistent between 2011 and 2017 (13 823 vs 13 674 respectively) but decreased by 12% for influenza‐like illness case‐patients (16 210 vs 14 477). Nine (50%) gained capacity to lineage‐type influenza B. The number of countries reporting each week to WHO FluNet increased from 15 (83%) in 2011 to 17 (94%) in 2017.

**Conclusions:**

Despite declines in external surveillance funding, ANISE countries gained additional laboratory testing capacity and continued influenza testing and reporting to WHO. These gains represent important achievements toward sustainable surveillance and epidemic/pandemic preparedness.

## 1 INTRODUCTION

Although sentinel surveillance in African countries for viral respiratory infections such as influenza is important for prevention and control, funding for such activities has steadily decreased making its sustainability uncertain. Africa has a higher influenza‐associated mortality burden than other regions. This is important as few African countries routinely vaccinate against influenza and or treat severe respiratory illnesses empirically with antivirals during the influenza seasons.^1^, ^2^ Much of Africa's population is low and middle income and have substantial prevalence of underlying medical conditions^3^, ^4^, ^5^ and limited access to health care, increasing the risk of severe complications as a result of influenza illness.^6^ Only 3 of Africa's 54 countries have government‐subsidized seasonal influenza vaccination programs.^7^ Nevertheless, more African countries have influenza vaccines available through the private sector, are evaluating the potential value of influenza vaccination,^8^, ^9^, ^10^ or are introducing publicly available influenza vaccines among key risk groups. In addition to nascent influenza vaccination programs, some countries in Africa also treat severe influenza illnesses during influenza epidemics and pandemics with empiric antivirals, and/or deploy non‐pharmaceutical interventions to prevent contagion during epidemics (eg, respiratory hygiene, social distancing, and hand washing campaigns).^5^, ^11^, ^12^, ^13^ The impact of these interventions is optimized by their timely deployment immediately before the anticipated start of epidemics.

Given the value of influenza surveillance for seasonal epidemic and pandemic mitigation, international agencies and governments provided substantial financial and technical resources to build global influenza surveillance capacity at the turn of the century.^14^ Much investment in capacity‐building in the early 2000s occurred in Africa which had a dearth of surveillance and a disproportionate disease burden. As a result, influenza surveillance rapidly improved throughout Africa during the peri‐pandemic period. The strengthening of surveillance allowed countries to better define their epidemic periods and, as a secondary benefit, estimate the burden of respiratory illnesses attributable to influenza.^15^, ^16^, ^17^, ^18^, ^19^, ^20^, ^21^ Investments were higher during the initial years of grants to encourage rapid capacity‐building and operationalization of surveillance, resources then tapered off with countries assuming greater technical and financial responsibility of their systems. For example, the US Centers for Disease Control and Prevention (CDC) funding to 15 African nations, through cooperative agreements, peaked during 2006 and decreased by approximately 50% in the subsequent decade.

The rapid investment and gradual divestiture strategy rapidly built sustainable influenza surveillance capacity in the Americas,^14^ but it is unclear if this strategy has also been effective in Africa. The African Network for Influenza Surveillance and Epidemiology (ANISE) is a regional consortium of subject matter experts seeking to improve surveillance in Africa. Most African countries who are members of ANISE have previously used their influenza‐like illness (ILI) and/or severe acute respiratory infection (SARI) or other respiratory disease surveillance systems to identify and test for the Middle East respiratory syndrome (MERS)^22^ and severe acute respiratory syndrome coronavirus 2 (SARS‐CoV‐2). Indeed, these platforms have been critical to the 2019 coronavirus disease (COVID‐19) pandemic response in Africa.^23^, ^24^ Given the importance of these platforms, we sought to evaluate influenza sentinel surveillance function in Africa from 2011 to 2017,^5^ after external funding to surveillance in Africa started to decrease and determine if initial investments in capacity‐building led to sustainable influenza surveillance.

## 2 METHODS

### 2.1 Study population

During the sixth ANISE^25^ meeting in Antananarivo, Madagascar in 2018, we invited ANISE member states to participate in Influenza Surveillance in Africa (InSAFRO), a survey to assess surveillance systems in Africa and their participation in the World Health Organization's (WHO) Global Influenza Surveillance and Response System (GISRS). ANISE is a network of laboratorians, epidemiologists, public health officials, physicians, veterinarians, researchers and policy makers who collaborate to strengthen capacity for respiratory virus surveillance and research in Africa. ANISE promotes the use of standardized protocols for surveillance of respiratory illnesses to generate evidence for public health interventions and policy.

### 2.2 Data collection

We used a standardized survey to gather information about sentinel surveillance practices, real‐time reverse transcription polymerase chain reaction (rRT‐PCR) capacity, the number of samples tested, and the number positive for influenza by week and age group during 2011‐2017. Influenza type, subtype, and lineage data were collected, if available. To explore whether influenza surveillance networks had expanded or contracted during the study period, we also gathered information about the type of surveillance conducted (ie, for ILI and/or SARI), and number of active sites. We assessed whether the case definitions reported by the survey matched WHO‐recommended ILI and SARI case definitions.^26^ Data were collected through the InSAFRO survey about national surveillance operational costs and funding sources. We reviewed reported avenues for regular dissemination of surveillance data in country to determine whether the generated influenza surveillance data were used to inform influenza prevention and control measures (eg, launching risk communication and vaccination campaigns). Finally, we surveyed participants about national vaccine policies or guidelines that could benefit from influenza surveillance.

### 2.3 Additional data collection

We also gathered information about influenza test results reported to the GISRS platform FluNet [40] and about samples shared with WHO Collaborating Centers (CCs). We reviewed published and unpublished burden of disease estimates solely to estimate the number from InSAFRO countries (ie, not to extract findings for a meta‐analysis); this review was conducted until September 2019. We summarized findings by World Bank income classification and population size^27^ to estimate the representativeness of samples collected and tested.

### 2.4 Data analyses

To describe surveillance capacity during 2011‐2017, we summarized the number of sentinel sites and rRT‐PCR throughput capacity for laboratory testing by country. The collection of potential risk factors for SARI and in‐hospital deaths were also noted. We estimated the relationship between annual rates of samples tested and positives by WHO transmission zone. We repeated this for each country during 7 years of surveillance using a generalized estimating equation method with repeated‐measures Poisson regression where the dependent variables were counts of influenza‐positive detections and samples tested for each year of the study and the independent variable WHO transmission zone.^28^ We explored the association between surveillance funding source and World Bank income classification status using Fisher's exact test to determine if there were non‐random associations between income classification and funding source (eg CDC, WHO, or Government) as categorical variables. We also plotted the number of samples tested and the proportion of influenza detections among ILI and SARI case‐patients during the 7‐year surveillance period by age group to determine changes in the amount of testing by age group. We used a linear regression to identify potential trends in the number of influenza viruses shared with WHO CCs and samples reported to FluNet over time. Lastly, we used Mann‐Whitney Rank Sum test to explore whether countries that used influenza vaccines in the private or public sector were more likely to report a greater number of influenza tests results to FluNet. Analyses were performed using SAS^®^ 9.4 and StataCorp 2019 Version 16.

### 2.5 Ethics and funding

This project was deemed a program evaluation and received a non‐research determination from the CDC IRB because it does not use data from human subjects; its primary intent was to determine the fitness and capacity of influenza surveillance to serve participating countries. The data collection forms and templates were reviewed and approved by the ANISE working group. Collaborating institutions received official invitation letters requesting information about their surveillance systems. This project was supported by the ANISE executive committee and funded by CDC.

## 3 RESULTS

### 3.1 Characteristics of surveillance systems

**FIGURE 1:**
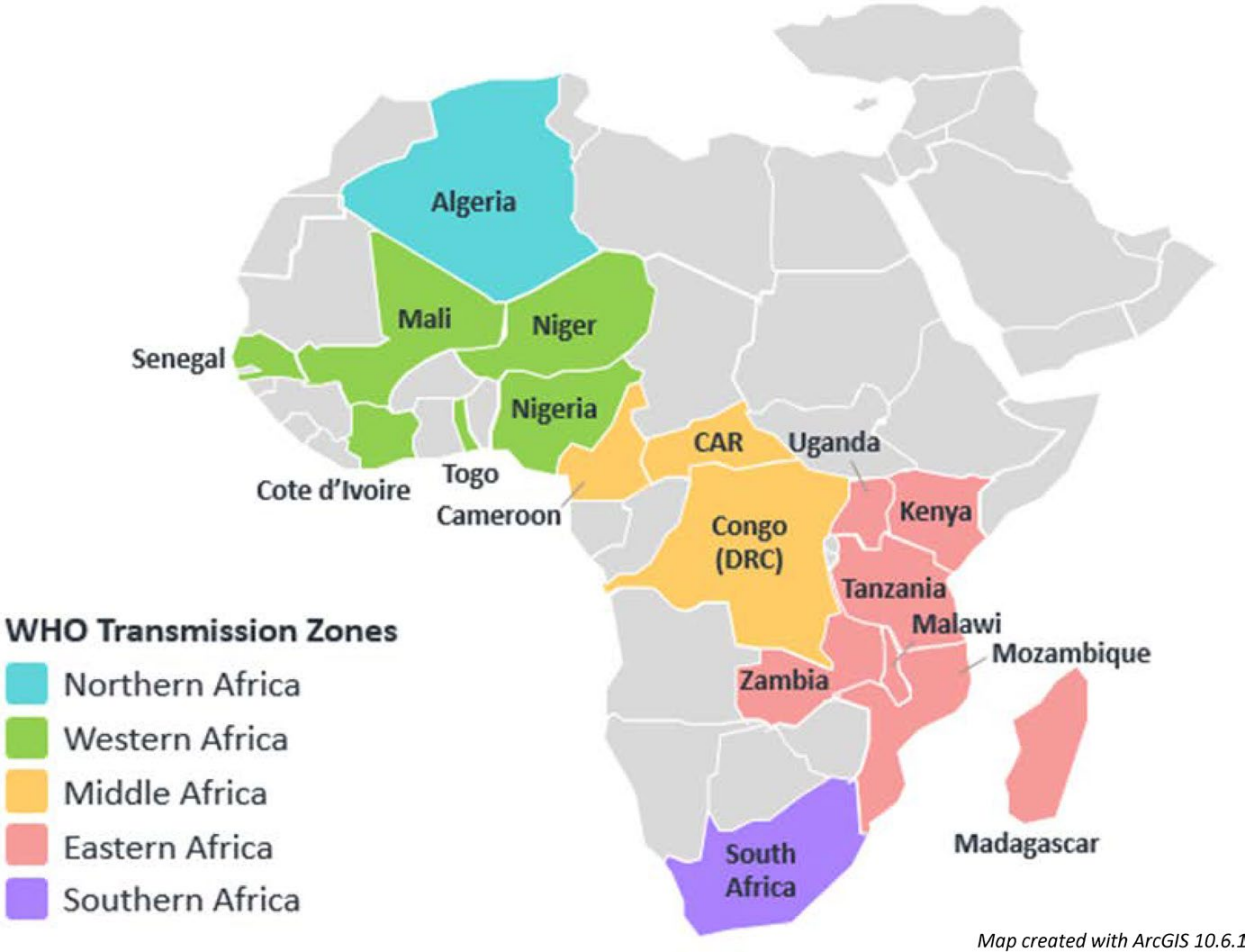
Countries participating in the influenza surveillance in Africa analysis

Twenty‐two (92%) of 24 ANISE members attended the 2018 annual meeting; 18 (81%) of these 22 agreed to participate in InSAFRO. These 18 countries have a cumulative population of 710 751 471 which represents 56% of Africa's total population (Table 1). Four countries did not contribute data for analysis: two did not attend the 2018 ANISE meeting and two were unable to complete the survey in time for analyses. On average, participating countries had conducted 13 years of influenza surveillance (range: 5‐41 years); South Africa and Madagascar had conducted surveillance the longest among the countries (ie, >30 years) (Figure 1).

**Table 1:** Characteristics of the influenza surveillance systems in participating countries 2011‐2017

WHO transmission zone	Country	^a^Population N (% of Africa), 2017	Year surveillance started	WHO‐designated National Influenza Center 2017	^d^EQAP (%)	Number of sentinel sites in 2017		Cumulative ILI specimens tested & % positive for influenza n/N; (%)	Cumulative SARI specimens tested & % positive for influenza (n/N; (%)	Mean annual specimens tested & % positive for influenza (n/N; (%)
						Influenza‐like illness	Severe acute respiratory infection	ILI	SARI	ILI & SARI
Northern	Algeria^b^, ^c^	41 398 198 (9)	2011	Y	100	15	1	3361 (27)	1096 (35)	637 (29)
Southern	South Africa^b^, ^c^, ^e^	56 717 156 (5)	1984	Y	89	3	8	9967 (9)	27 191 (4)	5308 (5)
Western	Côte d'Ivoire^b^, ^c^, ^e^	24 294 750 (2)	2007	Y	91	14	11	11 048 (10)	2927 (5)	1996 (9)
Western	Democratic Republic of Congo^e^	81 339 988 (8)	2008	N	92	11	9	8734 (6)	5755 (5)	2070 (6)
Western	Mali^e^	18 541 980 (2)	2014	N	73	5	3	2677 (12)	496 (8)	453 (11)
Western	Niger	21 477 348 (2)	2009	N	97	7	7	2103 (7)	2611 (6)	673 (6)
Western	Nigeria^e^	190 886 311 (18)	2009	N	99	4	4	7915 (7)	1627 (4)	1363 (6)
Western	Senegal	15 850 567 (1)	1996	Y	99	15	2	11 715 (13)	266 (13)	1720 (13)
Western	Togo^e^	7 797 694 (1)	2010	N	98	2	4	3996 (12)	556 (8)	650 (12)
Eastern	Kenya^e^	49 699 862 (5)	2006	Y	98	7	7	2476 (9)	17 194 (6)	2809 (7)
Eastern	Madagascar^c^, ^e^	25 570 895 (2)	1978	Y	97	54	18	8198 (23)	1771 (17)	1424 (22)
Eastern	Malawi	18 622 104 (2)	2011	N	85	1	2	1306 (12)	5415 (7)	960 (8)
Eastern	Mozambique^e^	29 668 834 (3)	2013	N	96	1	2	155 (14)	2582 (4)	390 (4)
Eastern	Tanzania^e^	57 310 019 (5)	2008	Y	97	13	12	4255 (9)	9130 (6)	1912 (7)
Eastern	Uganda^e^	42 862 958 (4)	2007	Y	100	6	8	9403 (11)	7705 (8)	2444 (9)
Eastern	Zambia^e^	17 094 130 (4)	2008	Y	99	2	4	4643(7)	4521 (6)	1309 (5)
Central Africa	Cameroon^c^	24 053 727 (2)	2007	Y	97	24	3	8347 (16)	1344 (13)	1384 (15)
Central Africa	Central African Republic	4 659 080 (0)	2008	Y	‐	7	1	7169 (4)	0 (–)	1024 (4)
Total		710 751 471 (56)	1978‐2014	55%	73‐ 100	11 (1‐54)	6 (1‐18)	107 520 (11)	92 181 (8)	28 529 (9)

^a^ Data populated with World Bank 2017 population data. Population for Middle East/North Africa used as denominator for Algeria.

^b^ Influenza vaccine policy or guidelines available.

^c^ Seasonal Influenza vaccine available in the public sector.

^d^ External Quality Assessment Panels (EQAP) data are averaged from 2011‐2017.

^e^ CDC‐funded countries.

InSAFRO countries collected influenza surveillance data through 187 ILI and 104 SARI sentinel sites. On average, countries had 11 (range: 1‐54) ILI and 6 (range: 1‐18) SARI sentinel sites. Ten (56%) of the 18 countries used the ILI and 14 (78%) used the SARI case definitions recommended by WHO.^26^ The eight countries that modified their ILI case definition and the four countries that modified their SARI case definition each identified cases with onset of fever within 7 days of illness onset rather than the WHO recommended 10 days.^26^

On average, countries tested a cumulative 28 529 specimens a year (Table 1). The number of ILI samples decreased by 12% from 16 210 in 2011 to 14 477 in 2017 while the number of SARI samples tested remained similar at approximately 14 000 specimens. Most (80%) of the 18 countries collected data about case‐patients' age and preexisting medical conditions. More than half of the samples collected were from children aged <5 years (ie, 53%, 95% CI: 51‐54 from ILI and 61%, 95% CI: 57‐65 from SARI). Few samples were from persons aged ≥65 years (1%, 95% CI: 1‐2 from ILI and 3%, 95% CI: 3‐3 from SARI). Only 10 (56%) of the 18 countries documented in‐hospital outcomes (eg death, recovery, and transfers) for SARI case‐patients.

InSAFRO countries spanned four WHO influenza transmission zones^28^; the Northern [Algeria] countries contributed a cumulative annual average of 636 specimens, the Western zone [Cote d'Ivoire, Democratic Republic of Congo, Mali, Niger, Nigeria, Senegal, Togo] 8926 specimens, the Eastern zone [Kenya, Madagascar, Malawi, Mozambique, Tanzania, Uganda, Zambia] 11 248 specimens, the Central zone [Cameroon, Central African Republic] 2408 specimens, and the Southern zone [South Africa] zone 5308 (*P* ≤ .0001). Based on a Poisson regression model no evidence of a difference was seen in the rate of samples tested per million persons per year between transmission zones among surveyed countries: Central zone (4.7/1 000 000‐y), Eastern zone (2.1/1 000 000‐y), Northern zone (2.2/1 000 000‐y), Southern zone (2.5/1 000 000‐y) and Western zone (1.1/1 000 000‐y, *P* ≤ .3).

**FIGURE 2:**
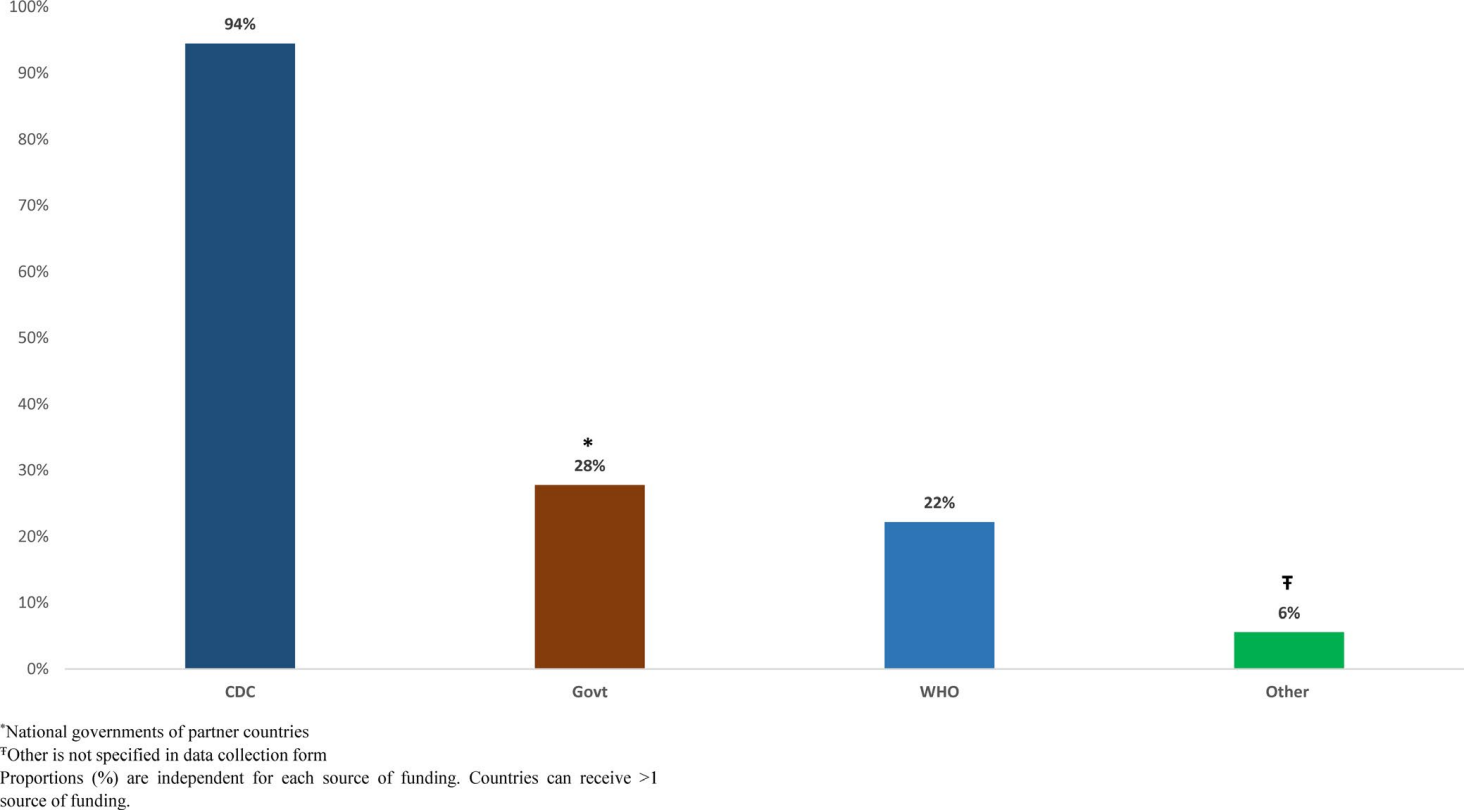
Funding sources for influenza surveillance in Africa member countries, 2011–2017

**FIGURE 3:**
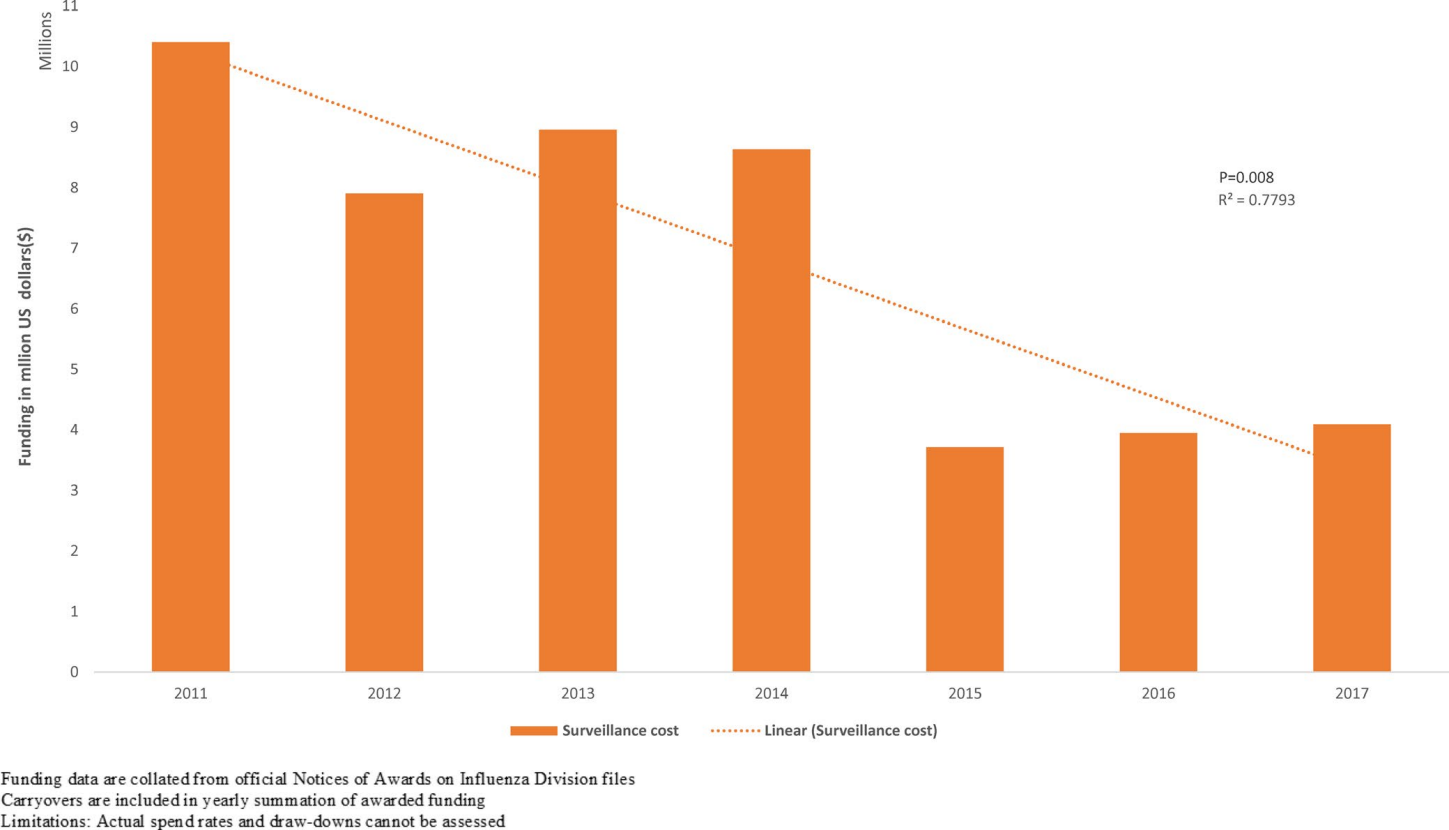
Funding trend in surveillance allocation from 2011 to 2017

Countries reported that influenza surveillance cost approximately between $10 000 and $1 267 280 to operate each year (median $105 000). All 18 countries paid for surveillance with funds from multiple agencies (Figures 2 and 3). We found no associations between the type of external funding and World Bank income classification. Seven (39%) of the 18 countries conducted influenza surveillance as part of an integrated disease surveillance system for improved detection and response to leading causes of illness.

#### 3.1.1 Laboratory capacity

Countries tested a median of 1373 (interquartile range [IQR]: 745‐1975) specimens through rRT‐PCR per year, of which on average 9% tested positive for influenza (range 7‐41%). The proportion of samples tested for influenza was higher among ILI (mean 58%, range: 54‐60) versus SARI (42%, range 34‐46) cases (*P* ≤ .002). All InSAFRO countries had rRT‐PCR capacity for laboratory testing. All countries subtyped influenza A and nine (50%) gained the ability to lineage‐type influenza B during 2011‐2017. Seventeen (94%) of the 18 InSAFRO countries scored a mean of (95%, range: 88‐98) on WHO external quality assurance panels ^29^ (Table 1). In 2011, there were eight National Influenza Centers (NIC) (44%) among the 18 countries; with Tanzania, Zambia, and Kenya subsequently gaining NIC status during the 2011‐2017 (61%) period.

#### 3.1.2 Reporting & contribution to GISRS

During 2011‐2017, the number of InSAFRO countries reporting to WHO FluNet increased from 15 (83%) to 17 (94%); all of which also had ≥4 consecutive years of year‐round surveillance by the end of the survey period. On average, the surveyed countries reported a greater number of specimens (21%) on WHO FluNet than were reported to our survey. Annually, InSAFRO countries also shipped in aggregate, an average of 728 influenza viruses to WHO CCs for virus characterization and strain selection for candidate vaccine viruses, a number which remained approximately the same during the study period.

While 11 (61%) of the 18 countries used influenza vaccines in the private sector and 5 (27%) in the public sector, only 3 (17%) had publicly available national guidelines or policies about the use of seasonal influenza vaccines. Seven (38%) had published or unpublished estimates of the burden of respiratory illnesses attributable to influenza that could be used to explore the value proposition of influenza vaccines.^4^, ^15^, ^16^, ^17^, ^20^, ^21^, ^30^ During the study period, countries with influenza vaccines in the private sector reported an average of 27 797 samples to FluNet vs 13 202 among those without vaccines in the private sector (*P* < .0001); data about vaccine use in the private sector for Malawi and Mozambique were unavailable for this analysis.

## 4 DISCUSSION

### 4.1 Surveillance and laboratory capacity

Our results suggest that following substantial investment in capacity‐building at the turn of the millennium, most of the 18 African countries we evaluated sustained or strengthened influenza surveillance and laboratory diagnostic capacity, routinely reported findings to WHO, and shared samples with WHO CCs. In 2012, Radin et. al reported a tenfold increase from 4623 specimens in 2006 to 44 763 in 2010 among 15 African countries, 9 of which overlap with our survey countries.^3^ Our findings suggest most InSAFRO participants have continued to strengthen surveillance in the decade after the *Radin* report.

All surveyed countries had the capacity to test influenza specimens through rRT‐PCR, which helped them to maintain situational awareness about respiratory viruses in their respective countries. Although the number of sentinel surveillance sites has not significantly increased from 2012, possibly in an effort to improve the cost‐benefit of surveillance, there was an overall increase in sampling and testing. Sampling for SARI respiratory increased especially among children aged 0‐4 years (Figure S2). The overall increase in SARI sampling, which was accompanied by a modest decrease in ILI sampling, might be attributed to an emphasis by the global community to strengthen severe respiratory illnesses surveillance to compensate for the perceived shortcomings of surveillance to track severe illnesses during the 2009 pandemic.^31^, ^32^

### 4.2 Participation in GISRS and WHO CCs

Investment in surveillance capacity‐building resulted in sustained reporting of virus activity to GISRS and shipment of specimens to WHO CCs, even after a 37% decrease in external funding for such activities. All countries contributed influenza viruses to one or more WHO CCs during the northern and southern hemisphere Vaccine Composition Meeting for vaccine candidate virus selection. Although specimen testing increased among participating countries, the number of positive viruses shared with WHO CCs by transmission zones remained similar throughout the study period (Figure S5). Although we suspect additional specimens were not requested by the CCs because of limitations in the number that CC's can characterize. There may be value in setting benchmarks for the minimum and maximum number of influenza specimens collected within the beginning, middle, and end of epidemics that should be shipped quarterly to WHO CCs assuming year‐round transmission. Such benchmarks might allow countries to ensure that they share timely specimens and maximize the chances that influenza viruses identified within their country will be adequately represented in vaccine formulations.

In addition to demonstrating sustained or increased testing capacity during 2011‐2017, our survey showcases the increase in the number of NICs in Africa and their newly attained ability to lineage‐type influenza B. NICs are critical to the sustainability of surveillance because they orchestrate laboratory surveillance within countries, provide support to subnational laboratories, and liaise with WHO and its CCs. NICs do proficiency testing for subnational laboratories, test clinical specimens for influenza, identify and further characterize viruses, and report findings to WHO through FluMart. NICs also collect specimens for shipment to CCs either emergently, when they identify what might be novel viruses, or quarterly to inform vaccine strains selection. This is evident during the current COVID‐19 pandemic where NICs are the underpinning for the rollout of testing for SARs‐CoV‐2.^23^, ^24^, ^33^

In 2018, the WHO revised the Terms of Reference (TOR) for NIC status to exclude the requirements for national laboratories to isolate influenza viruses. This shift in TOR recognized that some CCs prefer that countries send influenza‐positive specimens for rapid sequencing first rather than delay shipment with virus isolation attempts. In Africa, virus isolation requirements frequently held back national laboratories from achieving WHO NIC designation; we anticipate that more countries in Africa will now be able to attain WHO NIC designation because of the relaxed TOR.

### 4.3 Funding and expansion of surveillance

Despite reduction in external contributions, influenza surveillance seemed sustainable during the past decade. Thirteen countries received funds through US CDC cooperative agreements and indirect financial support from other partners (Figure 2). During 2010‐2019 two new countries were awarded capacity‐building funds to establish their national surveillance programs by the US CDC, five moved from capacity‐building cooperative agreements (on average 450 000 USD during a 5‐year period) to maintenance cooperative agreements (on average 250 000 USD) with a cumulative decrease in funding of 1 461 020.00 USD (37%). Two other countries moved to a sustainability cooperative agreement, and received on average 50 000 USD, to support their established surveillance activities.

Despite notable national and institutional commitments, surveillance systems in Africa still rely largely on external funding to sustain or increase surveillance capacity. All countries were also beneficiaries of the CDC‐funded International Reagent Resource, which provided registered countries with the reagents for the surveillance of novel and emerging influenza strains at no cost. In addition, WHO, Institut Pasteur, and national governments supported influenza surveillance and regional capacity strengthening to respond to outbreaks and for pandemic preparedness.

### 4.4 Vaccines and policy

Seasonal influenza vaccines are available in African countries, however public sector access remains limited. Influenza vaccines were available in the private and public sector in two‐thirds of the 18 surveyed countries and, although more than a third had influenza disease burden estimates, less than one in five countries had publicly available national vaccine guidelines or policies. In countries using influenza vaccines, these were typically available in pediatric health centers, private pharmacies, and embassies except for two countries (South Africa and Madagascar) where influenza vaccines were licensed, offered routinely at the point of care, and recommended in the public sector. In South Africa, for example, free seasonal influenza vaccines were available in all public primary care facilities with a limited stock of approximately 1 000 000 doses for use among persons at high risk of influenza complications. Of note, countries that used influenza vaccines in the private sector reported, on average, a higher number of samples to WHO FluNet.

Our survey suggests that a disproportionally high percent of specimens from InSAFRO countries came from children aged <5 years. Although 16% of the 2019 census population for countries is aged <5 years, children aged <5 years represented 61% of ILI and 53% of SARI specimens. Our finding is consistent with that of other studies in Africa including Zambia where 60‐80% of SARI samples were from young children.^21^ The large percent of children which comprise surveillance case‐patients might reflect underlying rates of severe respiratory illness among those in the extremes of age and health utilization patterns that focus scarce resource on children rather than older adults as noted in a handful of health utilization surveys.^17^, ^20^, ^21^, ^30^, ^34^, ^35^ Additional evaluations are needed to determine if the proportion of samples obtained from different age groups is representative of those who seek care at sentinel sites. Such evaluations might be useful because representative sampling was not included as a formal milestone for evaluation in CDC funding opportunity applications.

### 4.5 Limitations

The InSAFRO analyses were limited to surveillance data from 18 out of 54 WHO Member States in Africa. Nevertheless, these 18 countries comprised half of Africa's population and our findings might be generalizable to additional countries in Africa. We did not comprehensively quantify external funding countries received for surveillance. InSAFRO countries reported 21% more respiratory samples results to WHO FluNet than they reported to our survey, possibly because our survey allowed participants to report results once while FluNet allows countries to update backlogged results at any given time. We also did not enumerate the number of pediatric‐focused health facilities in order quantify the proportion of specimens from clients aged <5 that were collected from pediatric inpatient or outpatient health facilities. We did not explore exact amounts countries were awarded versus amounts spent in their cooperative agreement funds. Finally, our survey was not designed to explore how countries used surveillance findings for public health action.

## 5 CONCLUSION

Our InSAFRO survey suggests that investments in capacity‐building at the turn of the millennium led to sustainable influenza surveillance among African countries. Despite substantial decreases in external funding, countries tested more respiratory samples, especially among children with SARI, and reported to FluNet more than ever before. InSAFRO countries have continued to share samples with WHO CCs and to meaningfully participate in GISRS. During the study period, several countries successfully achieved WHO NIC designation, and some gained the ability to lineage test influenza B. These gains represent important achievements in seasonal and pandemic influenza preparedness. It will be important to continue to monitor capacity in the region and to observe if more countries use their gains in surveillance to strengthen vaccine programs and other respiratory virus mitigation and control measures.

## AUTHOR CONTRIBUTION

**Ledor Igboh:** Conceptualization (Equal); Data curation (Lead); Formal analysis (Lead); Methodology (Equal); Project administration (Equal); Writing‐original draft (Lead); Writing‐review & editing (Equal).**Meredith McMorrow:** Conceptualization (Equal); Data curation (Supporting); Formal analysis (Equal); Methodology (Equal); Validation (Equal); Writing‐review & editing (Equal). **Stefano Tempia:** Conceptualization (Supporting); Data curation (Supporting); Formal analysis (Supporting); Methodology (Equal); Validation (Supporting); Visualization (Supporting); Writing‐review & editing (Equal). **Gideon Emukule:** Conceptualization (Supporting); Data curation (Supporting); Formal analysis (Supporting); Methodology (Supporting); Validation (Equal); Writing‐review & editing (Equal). **Ndahwouh Nzussouo:** Data curation (Equal); Project administration (Equal); Writing‐review & editing (Equal). **McCarron Margaret:** Methodology (Supporting); Project administration (Supporting); Validation (Supporting); Writing‐review & editing (Supporting). **Thelma Williams:** Data curation (Supporting); Project administration (Supporting); Validation (Supporting). **Vashonia Weatherspoon:** Project administration (Supporting); Validation (Supporting); Writing‐review & editing (Supporting). **Fawzi Derrar:** Data curation (Supporting); Investigation (Supporting); Validation (Supporting); Writing‐review & editing (Supporting). **Richard Njouom:** Data curation (Supporting); Investigation (Supporting); Validation (Supporting); Writing‐review & editing (Supporting). **Emmanuel Nakouné:** Data curation (Supporting); Investigation (Supporting); Validation (Supporting); Writing‐review & editing (Supporting). **Daouda Coulibaly:** Data curation (Supporting); Investigation (Supporting); Validation (Supporting); Writing‐review & editing (Supporting). **Hugo Kavunga‐Membo:** Data curation (Supporting); Validation (Supporting); Writing‐review & editing (Supporting). **Mary Okeyo:** Data curation (Equal); Validation (Supporting), Writing‐review & editing (Equal). **Jean‐Michel Heraud:** Conceptualization (Supporting); Data curation (Supporting); Investigation (Supporting); Methodology (Supporting); Validation (Supporting); Writing‐review & editing (Supporting). **Ivan Mambule:** Data curation (Equal); Validation (Equal); Writing‐review & editing (Equal). **Sambe Sow:** Validation, Writing‐review & editing. **Almiro Tivane:** Data curation (Supporting); Validation (Supporting); Writing‐review & editing (Supporting). **Adamou Lagare:** Data curation (Supporting); Investigation (Supporting); Validation (Supporting); Writing‐review & editing (Supporting). **Adedeji Adebayo:** Data curation (Supporting); Validation (Supporting); Writing‐review & editing (Supporting). **Ndongo Dia:** Data curation (Supporting); Validation (Supporting); Writing‐review & editing (Supporting). **Vida Mmbaga:** Data curation (Supporting); Validation (Supporting); Writing‐review & editing (Supporting). **Issaka Maman:** Data curation (Supporting); Validation (Supporting); Writing‐review & editing (Supporting). **Julius Lutwama:** Data curation (Supporting); Validation (Supporting); Writing‐review & editing (Supporting). **Paul Simusika:** Data curation (Supporting); Validation (Supporting); Writing‐review & editing (Supporting). **Sibongile Walaza:** Data curation (Supporting); Validation (Supporting); Writing‐review & editing (Supporting). **Punam Mangtani:** Methodology (Supporting); Supervision (Supporting); Writing‐review & editing (Supporting). **Patrick Djomo:** Methodology (Supporting); Supervision (Supporting); Writing‐review & editing (Supporting). **Cheryl Cohen:** Conceptualization (Equal); Investigation (Supporting); Methodology (Supporting); Supervision (Supporting); Validation (Equal); Writing‐review & editing (Supporting). **Eduardo Azziz‐Baumgartner:** Conceptualization (Equal); Data curation (Supporting); Formal analysis (Equal); Investigation (Equal); Methodology (Equal); Supervision (Lead); Validation (Equal); Visualization (Equal); Writing‐review & editing (Equal).

## DISCLAIMER

The findings and conclusions in this article are those of the authors and do not necessarily represent the official position of the US Centers for Disease Control and Prevention (CDC).

### PEER REVIEW

The peer review history for this article is available at https://publons.com/publon/10.1111/irv.12818

ANISE Network Working Group Member listed in Appendix 1

## Supporting information

App S1

## Appendix 1

ANISE Network Working Group Member List

**Table:**

**#**	Name	Email Contact	Affiliation
1	El‐Aalia Gradi	aliapasteur@yahoo.fr	Institut Pasteur of Algeria
2	Chavely Gwlagys Monamele	monamele.chavely@yahoo.fr	Center Pasteur du Cameroon
3	Alexandre Manirakiza	alexandre.manirakiza@pasteur‐bangui.org	Institut Pasteur d'Bangui
4	Herve Kadjo	rvkdjo@yahoo.fr	Institut Pasteur de Cote d'Ivoire
5	Edith Nkwembe	edithnkwembe1@gmail.com	Institut National de Recherche Bio‐medicale, Democratic Republic of Congo
6	Norosoa Razanajavoto	norosoa@pasteur.mg	National Influenza Centre, Virology Unit, Institute Pasteur de Madagascar, Madagascar
7	Rosalia Kalani	Kalani2r@gmail.com	National Public Health Institute, Kenya
8	Anmol Kiran	akiran@mlw.mw	Malawi‐Liverpool Wellcome Trust Clinical Research Programme, Malawi
9	Adama Keita	admambyta@gmail.com	Central National Influenza Laboratory/ Ministry of Health, Mali
10	Neusa Nguenha	neusaguenha@gmail.com	Instituto Nacional de Saude, Mozambique
11	Mbayame Niang	Mbayame.Niang@pasteur.sn	Instituto Nacional de Saude, Mozambique
12	Florette Treurnicht	Florette.Treurnicht@nhls.ac.za	National Influenza Center, South Africa Centre for Respiratory Disease and Meningitis, National Institute for Communicable Diseases, South Africa
13	Edward Chentulo	edwardchents@gmail.com	University of Zambia Teaching Hospital, National Influenza Center, Zambia
14	Barnabas Bakamutumaho	bbarnabas2001@yahoo.com	Uganda Virus Research Institute, Uganda
15	Miriam Matonya	miriammatonya@gmail.com	National Reference Laboratory, Tanzania
16	Goumbi Kadade	goumbikadad@yahoo.fr	Ministry of Health, Niger
