# Supplementary material for: Influenza surveillance capacity improvements in Africa during 2011‐2017
Source: Influenza Other Respir Viruses. 2020 Nov 4;15(4):495–505. doi: 10.1111/irv.12818 (PMC8189239; doi:10.1111/irv.12818)
Supplement: Supplementary file 1 — App S1 [file IRV-15-495-s001.docx]

**
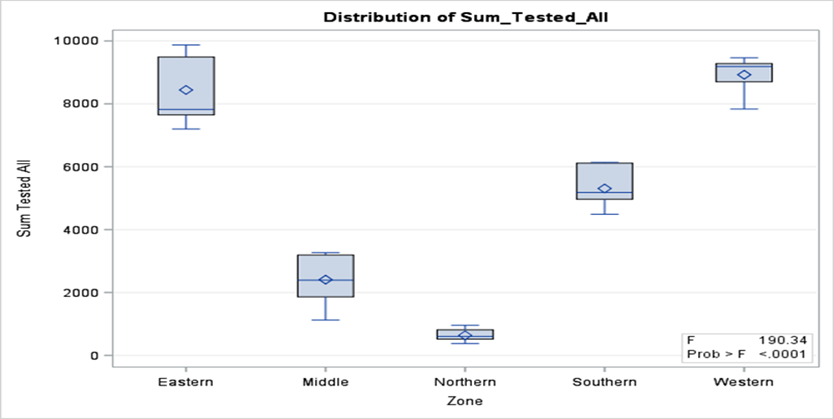
**

**Figure 1: Distribution of total influenza specimens tested by WHO transmission zones**

**
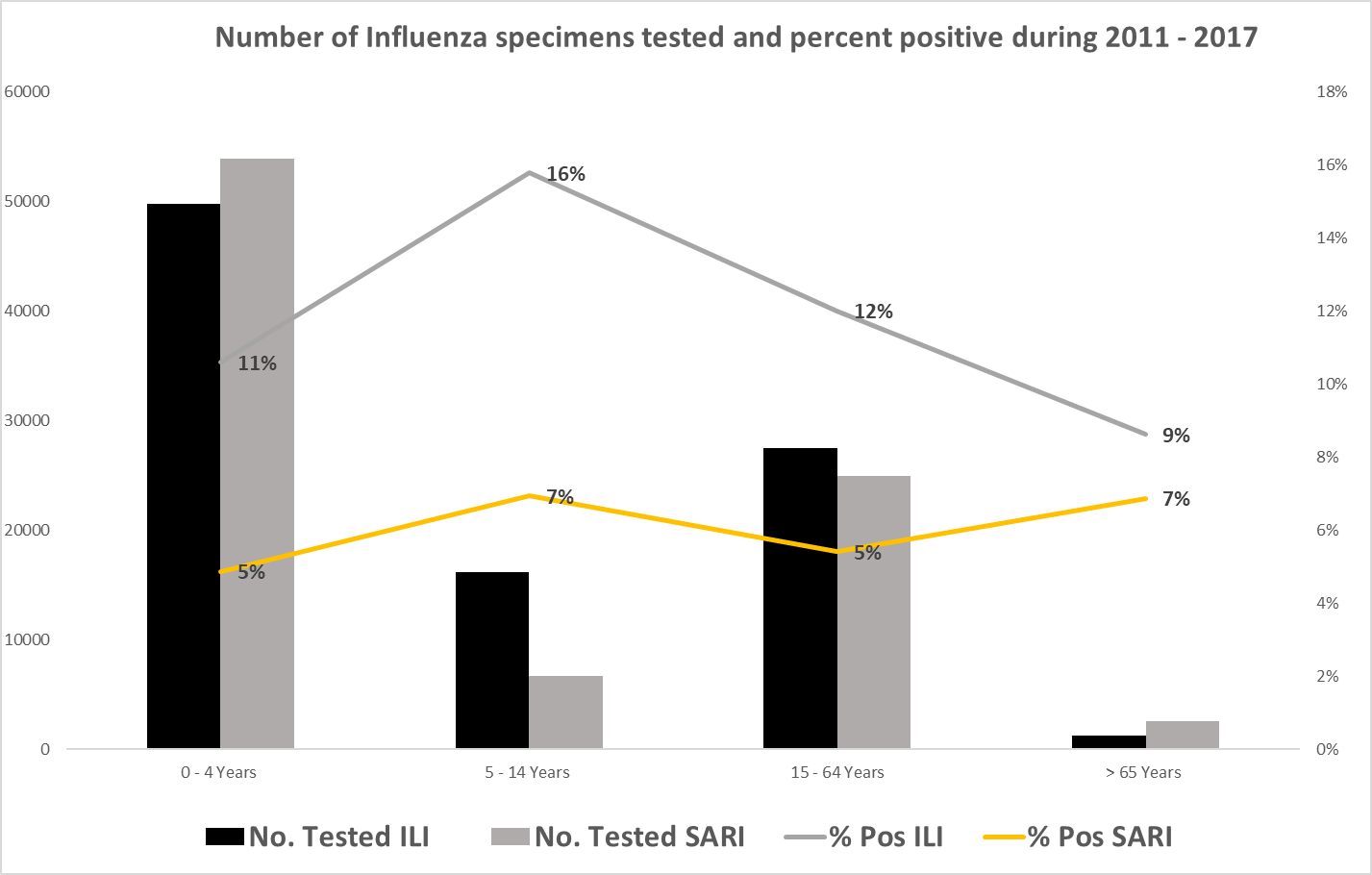
**

**Figure2: Number of Influenza specimens tested and percent positive during 2011 to 2017**

**Figure 3: Influenza specimens tested and proportion positive by age group for 2011**

**Figure 4: Influenza specimens tested and proportion positive by age group for 2017**

**Table 1: Proportion of influenza positives by type, subtype and lineage from 2011 - 2017**

| **Case Definition** | **Transmission Zone** | **Samples Received** | **Samples Tested** | **Positive Flu A** | **Positive Flu B** | **A(H1)** | **A(H3)** | **BYam** | **BVic** | **%Pos** | **% BYam** | **% BVic** | **%A(H1)** | **%A(H3)** |
| --- | --- | --- | --- | --- | --- | --- | --- | --- | --- | --- | --- | --- | --- | --- |
| ILI | Eastern | 30931 | 30428 | 4021 | 2846 | 816 | 2329 | 505 | 530 | 13% | 18% | 19% | 20% | 58% |
| ILI | Middle | 15516 | 15516 | 1555 | 1010 | 538 | 911 | 46 | 82 | 10% | 5% | 8% | 35% | 59% |
| ILI | Northern | 3387 | 3361 | 900 | 504 | 401 | 499 | 93 | 102 | 27% | 18% | 20% | 45% | 55% |
| ILI | Southern | 9973 | 9967 | 874 | 505 | 236 | 628 | 250 | 210 | 9% | 50% | 42% | 27% | 72% |
| ILI | Western | 48251 | 48248 | 4738 | 3219 | 2324 | 2322 | 491 | 575 | 10% | 15% | 18% | 49% | 49% |
| SARI | Eastern | 49207 | 48312 | 2983 | 1560 | 645 | 1516 | 64 | 34 | 6% | 4% | 2% | 22% | 51% |
| SARI | Middle | 1344 | 1344 | 171 | 49 | 82 | 64 | 4 | 4 | 13% | 8% | 8% | 48% | 37% |
| SARI | Northern | 1103 | 1096 | 380 | 62 | 331 | 49 | 20 | 5 | 35% | 32% | 8% | 87% | 13% |
| SARI | Southern | 27384 | 27191 | 1000 | 591 | 456 | 530 | 231 | 217 | 4% | 39% | 37% | 46% | 53% |
| SARI | Western | 14238 | 14238 | 764 | 442 | 418 | 305 | 95 | 118 | 5% | 21% | 27% | 55% | 40% |
| **Total** |  |  | **199701** | **17386** | **10788** | **6247** | **9153** | **1799** | **1877** |  |  |  |  |  |
| Abbreviations: ILI = influenza-like illness; SARI = severe acute respiratory infection. | | | | | | | | | | | | | | |

**Figure 5: Contributions of positive viruses to Collaborating Centers by WHO transmission zone**

**
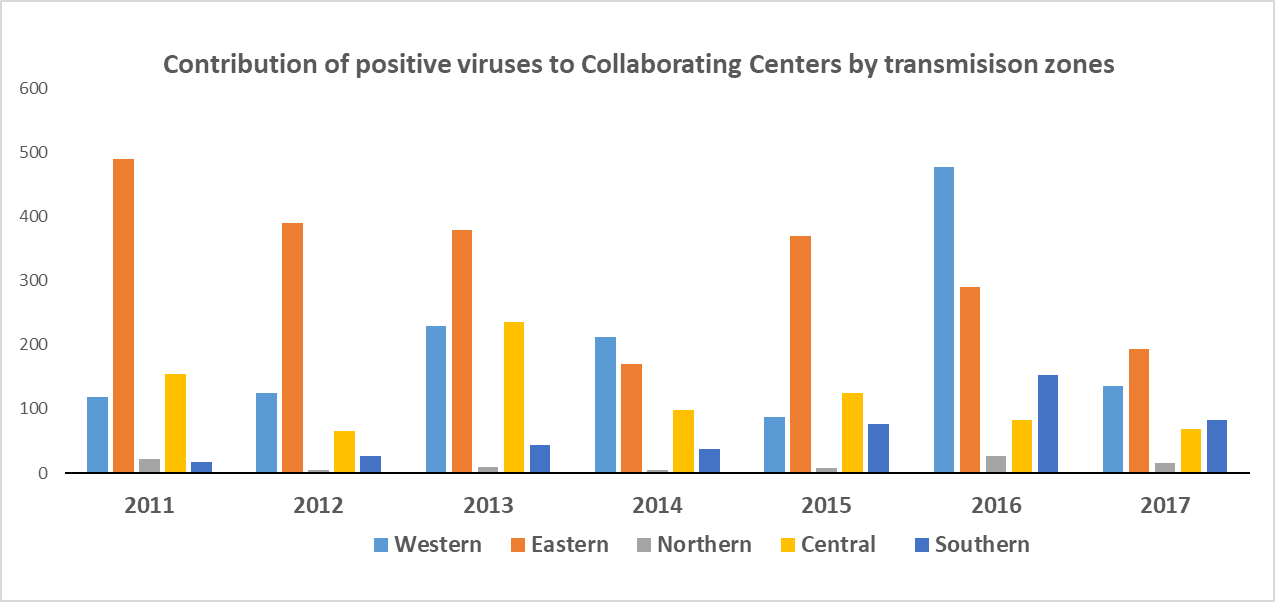
**

**
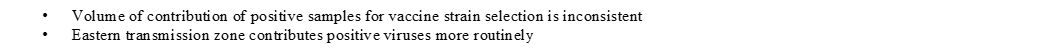
**

**Figure 5b (second option): Contributions of positive viruses to Collaborating Centers by WHO transmission zone**

**Figure 6: CDC funding trend for wages versus surveillance from 2011 - 2017**


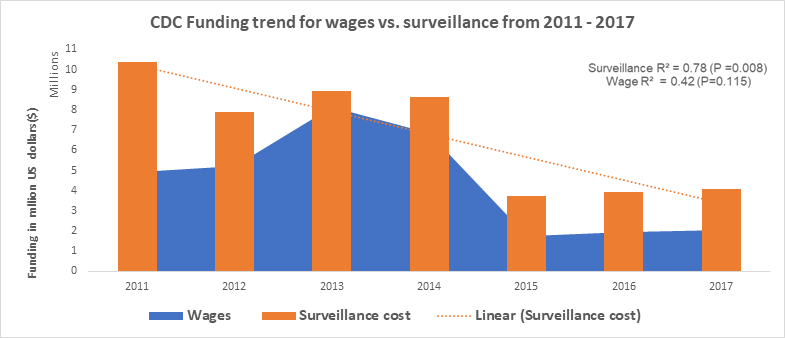


**
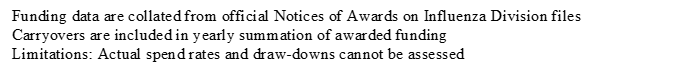
**
